# Sanitation at the Fair

**Published:** 1899-10

**Authors:** 


### SANITATION AT THE FAIR.

IN CONSIDERING the make-up of the medical corps for the Pan-American Exposition, the directors should carefully consider the appointment of a sanitary officer, whose duty it shall be to make as many daily inspections as he and the medical director deem necessary. Sanitary inspection means much. It means everything, and the man selected for this work will have a most responsible and most arduous position to fill. That being the case, he should be a man unquestionably fitted for the work, by reason of long experience in sanitary matters, and be thoroughly familiar with preventive medicine.

It is no reflection on the Pan-American officials to say they are not competent to make the appointment off-hand. The qualifications for the work to be done by the sanitary officer are peculiar, and because it is impossible for the laity to be thoroughly conversant with the scientific aspects of such an officer's duties the directors cannot be expected to make the best selection. This being so the JOURNAL would suggest that the selection be left to the Health Commissioner of Buffalo, Dr. Ernest Wende; that the city be requested to furnish a sanitary officer, and that the services of one of Dr. Wende's assistants in the work of the Health Department be given to the exposition by the city. The preliminary work would require only a portion of this officer's time, and would not materially interfere with his duty in the Health Department, it being largely of a consulting character. Then when the gates of the exposition were thrown open, the city could give him a leave of absence, and he would then be free to accept a salary from the exposition for the constant attention to sanitary matters, which safety would require. As a matter of self-protection it seems to the JOURNAL that the city would be willing to permit one of the Health Department staff to devote a portion of his time to the preliminary sanitary work.

